# Within- and cross-language repetition priming in verb generation: evidence from non-proficient Chinese-English bilinguals

**DOI:** 10.3389/fpsyg.2026.1857807

**Published:** 2026-07-29

**Authors:** Zhen Wang, Lin Fan, Lingyun Tian

**Affiliations:** 1 School of Foreign Languages and Cultures, Beijing Wuzi University, Beijing, China; 2 National Research Center for Foreign Language Education, Beijing Foreign Studies University, Beijing, China; 3 School of Foreign Languages, Harbin University of Science and Technology, Harbin, China

**Keywords:** Chinese-English bilinguals, implicit memory, processing level, repetition priming, verb generation

## Abstract

The present study aims to investigate the repetition priming within and across languages in verbs among non-proficient Chinese-English bilinguals, using a verb generation task. The impact of levels of processing on repetition priming was also examined. A total of 160 non-proficient Chinese-English bilinguals participated in the experiments, generating English verbs (Experiment 1) or Chinese verbs (Experiment 2) that corresponded to the same set of 50 translation-equivalent nouns across the two languages. The results revealed significant within-language repetition priming in both deep- and shallow-encoding conditions, whereas significant cross-language repetition priming was only observed in the deep-encoding condition. Within-language repetition priming was significantly greater compared to cross-language repetition priming. In addition, repetition priming within L2 English was significantly greater than that within L1 Chinese under deep-encoding condition. Cross-language priming showed a directional asymmetry, with stronger priming from Chinese to English than from English to Chinese. These results largely indicate that verb concepts are shared across the two typologically distant languages for non-proficient Chinese-English bilinguals. Notably, the repetition priming in verbs was modulated by levels of processing, with larger priming in the deep-encoding condition than in the shallow-encoding condition.

## 1 Introduction

The conceptual representation of translation-equivalent words in bilingual memory has been a topic of great interest in behavioral and neuroimaging research. A wealth of evidence has been accumulated to demonstrate the integrated conceptual representations of translation equivalents across languages in various language comprehension and production tasks, such as semantic classification task (e.g., Zeelenberg and Pecher, 2003; Li et al., 2009; Francis and Goldmann, 2011), verb generation task (Seger et al., 1999; de la Riva López et al., 2012), category exemplar generation task (Francis et al., 2010) and antonym generation task (Taylor and Francis, 2017). However, the existing language production studies providing direct evidence on this issue focused primarily on balanced or proficient bilinguals and languages that are typologically close, such as English and Spanish. Given the key role of typological distance in bilingualism research (Lee, 2022), it remains unclear whether the conceptual representations of translation-equivalent verbs are integrated or separated across two distant languages in non-proficient Chinese-English bilinguals. Most evidence for shared core conceptual representations of translation equivalents in episodic and semantic memory comes from experiments using noun concepts as stimuli (de la Riva López et al., 2012). Verbs occupy a core position in the lexicalization of events and states and are fundamental to understanding how humans represent and use information concerning such events and states in linguistic utterances (de Almeida and Manouilidou, 2015). Accordingly, an investigation into verb generation contributes greatly to understanding human language cognition. So far, only two studies have centered on the role of the levels of processing in verb generation task for monolinguals (Seger et al., 1999) and antonym generation task for balanced bilinguals (Taylor and Francis, 2017). The few available studies have shown some contradictory results, possibly due to differences in the language experience of the participants and the parts of speech of the stimuli. Thus, the impact of manipulations of levels of processing on repetition priming in non-proficient bilinguals’ verb generation awaits further investigation. The present study delves more deeply into this issue. Given these research gaps, it is of significance to investigate non-proficient Chinese-English bilinguals’ repetition priming in verb generation under different levels-of-processing manipulations. The investigation will be conducive to revealing the nature of the conceptual representations of translation-equivalent verbs in non-proficient bilinguals and the mechanisms underlying bilingual language processing.

## 2 Repetition priming in language production

Repetition priming, a measure to assess implicit memory, occurs when an initial presentation of a stimulus in the study phase exerts an effect on the performance of a participant’s response to that stimulus presented again in the test phase, and it is a well-established phenomenon in which participants typically respond faster and more accurately to a specific stimulus that has been studied recently than to an unstudied stimulus (Zeelenberg and Pecher, 2003). Repetition priming is thought to reflect lexical co-activation and conceptual representations across languages. The repetition priming paradigm is typically used to explore bilingual language representation and has been validated across a range of settings and populations in bilingual studies (e.g., Zeelenberg and Pecher, 2003; Li et al., 2009; de la Riva López et al., 2012; Taylor and Francis, 2017). As such, it is a valuable tool for investigating the cognitive processes underlying bilingual language use.

As an experimental instantiation of this paradigm, the picture naming task has often been used to investigate repetition priming effects during language production. Using this task, Hernandez and Reyes (2002) investigated within- and cross-language repetition priming in Spanish-English bilinguals. They found both within- and cross-language repetition priming effects, with an asymmetric pattern within language. Similarly, Francis et al.’s (2003) study of highly proficient Spanish-English bilinguals revealed an asymmetric pattern of within-language priming, but a symmetric pattern of cross-language priming in picture naming. Zhang and Wang (2005) observed repetition priming effects of Chinese-English bilinguals in a picture-naming study, with a greater magnitude of within-language repetition priming than cross-language repetition priming, and an asymmetry of cross-language repetition priming. Other researchers have also reported repetition priming in picture naming task, with slower responses in the non-dominant language relative to the dominant language (Francis and Sáenz, 2007; Francis et al., 2008). Two recent studies explored how prior comprehension exposure influences word production among Spanish-English bilinguals (Francis et al., 2025; Francis et al., 2025). The results revealed that comprehension exposure at the encoding phase yielded long-term learning effects on word production, and repetition priming arose from shared processing facilitation.

Additionally, different types of word production tasks, such as noun generation task, antonym generation task, verb generation task, were used to investigate the repetition priming effects. Fan et al. (2017) observed significant repetition priming in a noun generation experiment with Chinese-English bilinguals. Using adjectives other than nouns as stimuli, Taylor and Francis (2017) conducted an antonym generation experiment to explore the conceptual representations of adjectives among Spanish-English bilinguals. The results indicated significant within- and cross-language repetition priming in the deep-encoding condition, suggesting the shared adjective concepts across languages. Despite these studies, exceptionally few have investigated repetition priming in bilinguals’ verb generation. Seger et al. (1999) recruited 12 Spanish-English bilinguals to participate in a verb generation experiment and observed significant repetition priming. de la Riva López et al. (2012) further explored whether verb representations were shared or separated within and across languages among Spanish-English bilinguals. The results indicated that verb representations were integrated across languages and within-language repetition priming was stronger than cross-language repetition priming. The overall bilingual sample in Seger et al. (1999) was small (i.e., 12 participants), potentially leading to less reliable results. Moreover, these two existing bilingual studies focused only on proficient Spanish-English bilinguals (e.g., Seger et al., 1999; de la Riva López et al., 2012). There is a pressing need for further investigation of repetition priming in non-proficient bilinguals’ verb generation.

Repetition priming effects during word production can be modulated by different levels-of-processing manipulations. However, only two relevant studies are readily available (Seger et al., 1999, Exp. 5; Taylor and Francis, 2017). The levels of processing have been extensively investigated in dissociating implicit and explicit memory. The levels-of-processing effect refers to the finding that the subsequent memory performance on items at the test phase tends to be better in deep semantic processing compared to shallow perceptual, phonological, or orthographic processing at the encoding phase (Craik and Lockhart, 1972), indicating that the degree to which items are processed influences memory performance. Deep processing involves semantic analysis and word meaning access, such as semantic categorization and pleasantness rating, whereas shallow processing only involves structural analysis, such as syllable and rhyme judgments. The “levels of processing” framework (Craik and Lockhart, 1972) has been influential in the realm of memory research. While a great deal of evidence has indicated null effects of the levels-of-processing manipulations on implicit memory and their significant effects on explicit memory (e.g., Bowers and Schacter, 1990; Mahdavian and Kormi-Nouri, 2008), contradictory findings have been reported in verb generation and category exemplar generation tasks. Several experimental studies found that the priming magnitude in the semantic processing condition is larger than that in superficial phonological or orthographic processing condition (e.g., Hamann, 1990; Mulligan et al., 1999; Seger et al., 1999; Taylor and Francis, 2017), providing evidence for the impact of levels-of-processing manipulations on implicit memory performance. Therefore, the exact mechanisms underlying the levels-of-processing effect are not yet distinctly understood (Galli, 2014).

The relationship between bilingual language dominance and repetition priming has also been scrutinized. Most of the precursor research indicated that response times (RTs) in verb generation task (de la Riva López et al., 2012), noun generation task (Fan et al., 2017) and picture naming task (e.g., Francis and Sáenz, 2007; Francis et al., 2008) were longer for words in the non-dominant language than those in dominant language. Meanwhile, repetition priming effects during word production are sensitive to participants’ bilingual language dominance. Several previous studies demonstrated that repetition priming in noun generation (Fan et al., 2017) and picture naming (e.g., Francis et al., 2003; Francis et al., 2008; Francis et al., 2022) was weaker in the dominant language than in the non-dominant language. In a similar vein, evidence from word translation tasks revealed stronger repetition priming when responses were given in the non-dominant language (e.g., Francis et al., 2011). These effects can be elegantly explained by the Revised Hierarchical Model (RHM, Kroll and Stewart, 1994) and the frequency-lag hypothesis (Gollan et al., 2011). The RHM posits that the links of L1 lexical and conceptual representation are much closer as compared to those of L2. The frequency-lag hypothesis assumes that these effects arise because the words in the dominant language link closer to their corresponding concepts than those in the non-dominant language. Francis et al. (2022) also argue that the less well-learned words get greater benefits from repetition. Thus, it is not surprising that the larger repetition priming can be observed in the non-dominant language. To be noted, no significant differences between L1 and L2 repetition priming were found in de la Riva López et al.’s (2012) study, possibly due to their bilingual participants’ high level of proficiency in both L1 and L2. The Multilink model (Dijkstra et al., 2019) advocates that non-proficient and proficient bilinguals differ in language production since they exhibit distinct Resting Level Activation (RLA) in language brain regions. However, little empirical evidence from verb generation tasks has yet to support this theoretical claim. Therefore, it is reasonable to predict that the non-proficient bilinguals would exhibit greater effects of language dominance on verb generation RTs and priming than their proficient counterparts. This is one of the issues to be addressed in this study. If this prediction is empirically confirmed, the current results would, to a certain extent, offer further empirical evidence supporting the Multilink model (Dijkstra et al., 2019).

## 3 The present study

The current study has three goals. The first goal is to replicate the repetition priming previously observed in alphabetic languages and proficient bilinguals. The second one is to further compare different patterns of repetition priming, such as within-language repetition priming (e.g., L1-L1/L2-L2), cross-language repetition priming (e.g., L1-L2/L2-L1), and the magnitude of within-language repetition priming and cross-language repetition priming. Therefore, two parallel experiments were conducted, involving the English and the Chinese verb generation tasks, respectively. The third goal is to further assess the contribution of the levels-of-processing manipulations to repetition priming in verb generation, including the semantic processing in the deep-encoding condition and phonological or orthographic processing in the shallow-encoding condition.

### 3.1 Experiment 1: English verb generation

#### 3.1.1 Method

##### 3.1.1.1 Participants

Eighty Chinese-English bilinguals (61 females, 19 males, *M*_age_ = 18.0, range 17–20) were recruited from the first-year college students at Beijing Foreign Studies University to participate in Experiment 1. All of them signed informed consent forms and received a small monetary reward for their participation. This experiment was approved by the Research Ethics Board of Beijing Foreign Studies University. Participants were non-English majors with normal or corrected-to-normal visual acuity and did not pass the College English Test Band 4 (CET-4), a nationwide English proficiency test for Chinese college students. They reported Chinese as their first language (L1) and English as their second language (L2). Prior to the experiments, participants’ language profiles were assessed using a seven-point Likert-type scale (1 = unskilled, 7 = proficient). The descriptive statistics of participants’ self-reported items across experiments are shown in Table 1. They had studied English for 5 to 16 years (*M* = 11.1, *SD* = 2.24). Paired-samples *t*-tests revealed significant differences between Chinese and English proficiency in terms of listening [*t* (79) = 22.86, *p* < 0.001], speaking [*t* (79) = 26.69, *p* < 0.001], reading [*t* (79) = 21.28, *p* < 0.001] and writing [*t* (79) = 21.31, *p* < 0.001]. Drawing on experimental manipulations from existing research on non-proficient bilinguals (e.g., Li et al., 2009; Ma et al., 2020), and considering a notable proficiency difference between the two languages, the participants in the current study were categorized as non-proficient Chinese–English bilinguals.

**Table 1:** The descriptive statistics of self-reported items across experiments.

Items		Ex. 1 (*N* = 80)	Ex. 2 (*N* = 80)
Age		18.0 (0.63)	18.1 (0.50)
Years of learning English		11.1 (2.24)	10.7 (2.21)
Chinese	Listening	6.96 (0.25)	6.90 (0.38)
	Speaking	6.93 (0.38)	6.83 (0.61)
	Reading	6.88 (0.43)	6.85 (0.45)
	Writing	6.74 (0.69)	6.71 (0.70)
English	Listening	4.11 (1.09)	3.95 (1.07)
	Speaking	4.04 (0.91)	3.89 (1.02)
	Reading	4.50 (0.95)	4.55 (0.95)
	Writing	4.06 (0.99)	4.01 (1.00)

##### 3.1.1.2 Design

The experimental design was a within-subjects factorial design with two independent variables: encoding language (L1 and L2) and processing level (shallow and deep). An additional control condition was also included. Half of the words to be encoded were presented in Chinese and the other half in English. Words assigned to the control condition served as a baseline for measuring repetition priming and were not displayed at the encoding phase. The dependent variables were RTs and response accuracy (ACC). Shallow processing referred to orthographic processing, while deep processing referred to semantic processing. The language of verb generation at the test phase in Experiment 1 was English. Thus, the language manipulation was defined based on whether participants processed target words in English (within-language) or Chinese (cross-language) at the encoding phase.

##### 3.1.1.3 Materials

A total of 309 nouns were initially selected as candidate stimuli. Of these, 114 nouns were drawn from previous studies (Seger et al., 1999; de la Riva López et al., 2012), while the remaining 195 nouns were sourced from dictionaries or generated through brainstorming, based on their high consistency with corresponding verbs in L1 and L2. Subsequently, 49 nouns judged to be potentially unfamiliar to the participants were eliminated. The remaining 260 nouns were compiled into two counterbalanced questionnaires (130 English and 130 Chinese items per version) for a pilot study. The pilot study was conducted with 240 English as a Foreign Language (EFL) learners comparable to the participants in the present study. These EFL learners were divided into two equal groups, each receiving one version of the questionnaire, and were required to write down the first verb that came to their mind in response to each noun. Based on the consistency of verb responses in both language conditions, 50 nouns were ultimately selected as final stimuli. None of the selected nouns elicited identical dominant verb response.

The frequencies of L1 words were taken from Chinese Lexical Database (CLD, Sun et al., 2018), a 134.9-million-word corpus, while L2 word frequencies were sourced from the British National Corpus (BNC), a 100-million-word database of word tokens. The two separate databases were converted into the same size in order to compare the word frequencies of the two languages. Differences in frequency between L1 and L2 items were not significant at the encoding phase [*t* (38) = 1.063, *p* = 0.294] and test phase [*t* (48) = 0.981, *p* = 0.332]. No significant differences were found between shallowly studied and non-studied L1 words in terms of frequencies [*t* (13) = 0.527, *p* = 0.607] and strokes [*t* (13) = 0.124, *p* = 0.904]. Neither the differences in frequencies nor those in strokes were found between deeply studied and non-studied L1 words [*t* (13) = 0.735, *p* = 0.475; *t* (13) = 0.451, *p* = 0.660]. There were no statistical differences between shallowly studied and non-studied L2 words in terms of frequencies [*t* (13) = 0.071, *p* = 0.945] and length [*t* (13) = −0.082, *p* = 0.936]. Neither the differences in frequencies nor those in length were observed between deeply studied and non-studied L2 words [*t* (13) = 0.324, *p* = 0.751; *t* (13) = −0.227, *p* = 0.824]. Additionally, the frequencies of L1 and L2 target words used to make semantic relatedness judgments were also matched [*t* (18) = −0.411, *p* = 0.686]. Half of the L1 and L2 items were semantically related, and the other half was not semantically related. Thirty-six students (*M_age_* = 19.5, *SD* = 1.00), none of whom participated in the two experiments, assessed the materials of the semantic relatedness judgment task. The results indicated that the ACC rate in L1 and L2 semantic relatedness judgment varied from 91.67 to 100%. The mean ACC rate reached 98.05%, demonstrating the reliability of the materials in the deep-encoding task.

##### 3.1.1.4 Procedure

**Figure 1:**
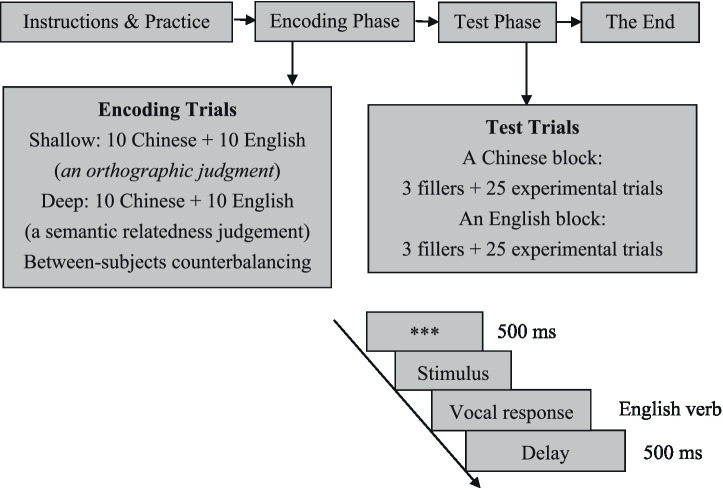
Flow chart of the experimental procedure.

The procedure of Experiment 1 is illustrated in Figure 1. Participants first received both written and oral instructions about the experiment. This was followed by a practice section to familiarize participants with the procedure.

The main experiment consisted of an encoding phase and a test phase. The encoding phase comprised a shallow-encoding task and a deep-encoding task. The shallow-encoding task was an orthographic judgment task where participants were asked to decide whether the component presented at the bottom of the screen was a part of the prime word at the top or not. The deep-encoding task was a semantic relatedness judgment task where participants decided whether the word at the bottom of the computer screen was semantically related with the prime word presented above as quickly as possible. Participants were randomly assigned to two groups to control key assignment effects. One group responded by pressing “j” for “Yes” and “f” for “No” with their left or right hand. For instance, if presented with the word “飞机” and “几” in the shallow condition, they were asked to make a “Yes” response. If presented with the word “oxygen” and “computer” in the deep condition, they had to make a “No” response. The other group used the opposite key assignment, with “j” representing “No” and “f” representing “Yes.” A between-subjects counterbalancing design was adopted across all participants. Each encoding condition (shallow and deep) consisted of two blocks, one in Chinese and one in English. Each block contained 10 trials, with stimuli presented one at a time. Both the sequence of stimuli and the language used in the encoding phase were counterbalanced between subjects using a Latin square matrix. Response ACC was recorded and used as an exclusion criterion for insufficient engagement with the encoding task.

The test phase began immediately after all encoding trials. It included a Chinese block and an English block. Each block consisted of 28 trials, including 3 fillers and 25 experimental trials with noun stimuli. Participants were required to produce the first verbs related to the nouns displayed one at a time on the screen in English. Each trial started with a 500-ms fixation asterisk, followed immediately by the target stimulus, which remained on the screen for an infinite time until a vocal response was recorded. Participants spoke out the first verb that came into their mind loudly, and a 500-ms delay was set before the next trial appeared. E-prime 3.0 was used for the stimuli display and data collection. Participants’ responses were recorded for each trial (if there was any response) via a Chronos box connected to a computer audio recorder. The experiment lasted approximately 20 min.

#### 3.1.2 Results of English verb generation

Eight participants were excluded from the analysis for failing to reach 90% accuracy in the encoding phase. Consequently, 72 participants were included in the analyses. If participants made a response that was not a verb, or was unrelated to the noun stimulus, or was not the expected one, it would be defined as a response error. Before calculating average RTs for each participant, response error trials (13.6%) and pass trials (10.5%) were removed. Mean RTs for correct trials were calculated after eliminating responses that were more than 3 SDs away from the mean. This procedure resulted in an average data loss of 1.8%. After data cleaning, 74.1% of trials were analyzed statistically with SPSS 22.0.

##### 3.1.2.1 Results of RTs

The mean RTs and priming of English verb generation are shown in Table 2. The results of single-sample *t*-tests indicated substantially significant within- and cross-language priming in the deep-encoding condition, *t* (71) = 7.832, *p* < 0.001, *t* (71) = 4.761, *p* < 0.001, respectively. There was also significant within-language priming in the shallow-encoding condition, *t* (71) = 5.381, *p* < 0.001, but no significant cross-language priming was observed, *t* (71) = 0.986, *p* = 0.327.

**Table 2:** Mean (SE) RTs and priming of English verb generation.

Measure	Control	Deep		Shallow	
		Within-language	Cross-language	Within-language	Cross-language
RT	2697.64	1908.28	2233.90	2227.38	2569.94
	(131.48)	(70.97)	(93.08)	(91.88)	(157.91)
Priming	—	789.36	463.74	470.26	127.70
		(100.78)	(97.41)	(87.40)	(129.45)

A 2 (encoding language) × 2 (processing level) repeated measures ANOVA was conducted on the mean priming data. The priming data were obtained by subtraction of the data in each condition from the data in the control condition. The analysis of priming data demonstrated a significant main effect of encoding language, *F* (1, 71) = 15.32, *p* < 0.001, η*_p_^2^ =* 0.177. Within-language priming was larger (629.81 ms) compared to cross-language priming (295.72 ms). The main effect of processing level was also significant, *F* (1, 71) = 27.34, *p* < 0.001, η*_p_^2^* = 0.278. The priming was stronger in the deep-encoding condition (626.55 ms) than in the shallow-encoding condition (298.98 ms). However, the interaction between encoding language and processing level was not significant, *F* (1, 71) = 0.013, *p* = 0.909, η*_p_^2^* = 0.000. Multiple paired-samples *t*-tests for post-hoc pairwise comparisons were conducted. The Bonferroni correction was applied to control familywise Type I error, with an adjusted significance threshold of *α* = 0.0125 across the four comparisons. Follow-up tests further revealed that within-language priming was reliably larger compared to cross-language priming in the deep-encoding condition, *t* (71) = −4.942, uncorrected *p* < 0.001, an effect that remained significant after Bonferroni adjustment. By contrast, this difference was not significant after correction in the shallow-encoding condition, *t* (71) = −2.353, uncorrected *p* = 0.021. Within- and cross-language priming in the deep-encoding condition were significantly greater than those in the shallow-encoding condition, *t* (71) = 4.219, uncorrected *p* < 0.001, *t* (71) = 2.937, uncorrected *p* = 0.004, satisfying the Bonferroni-adjusted significance threshold.

##### 3.1.2.2 Results of response ACC

The results of response ACC of English verb generation are presented in Table 3. The ACC rate of English verb-generation responses was assessed by dividing the number of correct trials by 10 trials per condition. The results indicated that within-language priming was significant in the deep-encoding [*t* (71) = 4.283, *p* < 0.001] and shallow-encoding conditions [*t* (71) = 2.559, *p* = 0.013]. Notably, there was significant cross-language priming in the deep-encoding condition, *t* (71) = 4.054, *p* < 0.001, but not in the shallow-encoding condition, *t* (71) = 1.428, *p* = 0.158.

**Table 3:** Mean (SE) ACC and priming of English verb generation.

Measure	Control	Deep		Shallow	
		Within-language	Cross-language	Within-language	Cross-language
ACC	0.70	0.80	0.79	0.77	0.74
	(0.02)	(0.02)	(0.01)	(0.02)	(0.02)
Priming	—	0.10	0.09	0.07	0.04
		(0.02)	(0.02)	(0.03)	(0.02)

The repeated measures ANOVA on the ACC rates and priming showed a main effect of processing level, *F* (1, 71) = 8.199, *p* = 0.006, η*_p_^2^* = 0.104. The ACC priming in the deep-encoding condition (0.09) was significantly greater than that in the shallow-encoding condition (0.05). No significant main effect of encoding language was observed, *F* (1, 71) = 2.464, *p* = 0.121, η*_p_^2^* = 0.034, nor was there a significant interaction between encoding language and processing level, *F* (1, 71) = 0.491, *p* = 0.486, η*_p_^2^* = 0.007.

### 3.2 Experiment 2: Chinese verb generation

#### 3.2.1 Method

##### 3.2.1.1 Participants

The participants in Experiment 2 were 80 non-proficient Chinese-English bilinguals (20 males) with a mean age of 18.1 years. All were first-year college students at Beijing Foreign Studies University, primarily from non-English majors. Written informed consent was obtained from each participant, and this experiment was conducted under the permission of the Research Ethics Board of Beijing Foreign Studies University. Participants had studied English for an average of 10.7 years (*SD* = 2.21), ranging from 5 to 15 years. Paired-samples *t*-tests indicated significant differences between Chinese and English language proficiency in listening [*t* (79) = 24.75, *p* < 0.001], speaking [*t* (79) = 23.75, *p* < 0.001], reading [*t* (79) = 20.60, *p* < 0.001] and writing [*t* (79) = 21.38, *p* < 0.001]. In line with the operational criteria for non-proficient bilinguals set in Experiment 1, all participants in this experiment were also identified as non-proficient Chinese–English bilinguals.

##### 3.2.1.2 Design, materials and procedure

Experiment 2 was identical to Experiment 1 in design, materials, and procedure, except that verb generation in the test phase was conducted in Chinese.

#### 3.2.2 Results of Chinese verb generation

The data trimming and analyses procedures of Experiment 2 were exactly the same as those described in the first experiment. Data from six participants were eliminated because their ACC rate was lower than 90%, leaving a total of 74 participants for the analyses. Pass trials (11.2%) and the trials that elicited error responses (7.6%) were excluded. For each participant, items with RT beyond 3 SDs above or below the mean (1.9%) were removed from the analysis. Descriptive statistics for mean RTs and ACC rate for the Chinese verb generation task are shown in Tables 4, 5.

**Table 4:** Mean (SE) RTs and priming of Chinese verb generation.

Measure	Control	Deep		Shallow	
		Within-language	Cross-language	Within-language	Cross-language
RT	1736.94	1491.84	1457.08	1658.57	1817.66
	(57.92)	(49.67)	(45.66)	(58.35)	(60.46)
Priming	—	245.10	279.86	78.37	−80.72
		(46.86)	(42.21)	(39.38)	(54.33)

**Table 5:** Mean (SE) ACC rates and priming of Chinese verb generation.

Measure	Control	Deep		Shallow	
		Within-language	Cross-language	Within-language	Cross-language
ACC	0.81	0.82	0.85	0.79	0.80
	(0.02)	(0.02)	(0.01)	(0.02)	(0.02)
Priming	—	0.01	0.04	−0.02	−0.01
		(0.02)	(0.02)	(0.02)	(0.02)

##### 3.2.2.1 Results of RTs

The mean RTs and priming of Chinese verb generation are given in Table 4. The results showed significant within- and cross-language repetition priming in the deep-encoding condition, *t* (73) = 5.230, *p* < 0.001, *t* (73) = 6.631, *p* < 0.001. In the shallow-encoding condition, within-language repetition priming was significant, *t* (73) = 1.990, *p* = 0.050, while cross-language priming was not significant, *t* (73) = −1.486, *p* = 0.142.

The results of the repeated measures ANOVA indicated that the main effect of processing level was significant, *F* (1, 73) = 82.89, *p* < 0.001. η*_p_^2^* = 0.532. Priming was considerably larger in the deep-encoding condition (262.48 ms) relative to the shallow-encoding condition (−1.17 ms). The main effect of encoding language was not significant, *F* (1, 73) = 3.09, *p* = 0.083. η*_p_^2^* = 0.041. Within-language priming (161.74 ms) was numerically stronger than cross-language priming (99.57 ms). The interaction between encoding language and processing level was significant, *F* (1, 73) = 11.78, *p* = 0.001. η*_p_^2^* = 0.139. In a similar vein, all pairwise comparisons were performed and adjusted via the Bonferroni method to control the familywise Type I error, yielding an adjusted significance threshold of *α* = 0.0125. Pairwise comparison test further showed that within-language priming was significantly larger than cross-language priming in the shallow-encoding condition, uncorrected *p* = 0.002, a contrast that remained statistically significant following Bonferroni adjustment. In contrast, no reliable difference was observed between within- and cross-language priming in the deep-encoding condition, uncorrected *p* = 0.407. Both within-language and cross-language priming effects in the deep-encoding condition were significantly greater than those in the shallow-encoding condition (both uncorrected *ps* < 0.001) and these two contrasts reached statistical significance following correction.

##### 3.2.2.2 Results of response ACC

Response ACC was measured in the same way as in Experiment 1, and mean ACC rates and priming are presented in Table 5. The results indicated that no significant within-language priming was observed in the deep-encoding and shallow-encoding conditions, (*ps* > 0.05). Moreover, cross-language priming was significant in the deep-encoding condition, *t* (73) = 2.173, *p* = 0.033, but was not significant in the shallow-encoding condition, *t* (73) = −0.436, *p* = 0.664.

A 2 (encoding language) × 2 (processing level) ANOVA was conducted on ACC priming. The main effect of processing level was significant, *F* (1, 73) = 8.106, *p* = 0.006, η*_p_^2^* = 0.100. The ACC priming in the deep-encoding condition (0.024) was greater than that in the shallow-encoding condition (−0.014). Nonetheless, the main effect of encoding language was not significant, *F* (1, 73) = 1.509, *p* = 0.223, η*_p_^2^* = 0.020, neither was the interaction between encoding language and processing level, *F* (1, 73) = 0.556, *p* = 0.458, η*_p_^2^* = 0.008.

### 3.3 Comparison of English and Chinese verb generation

**Figure 2:**
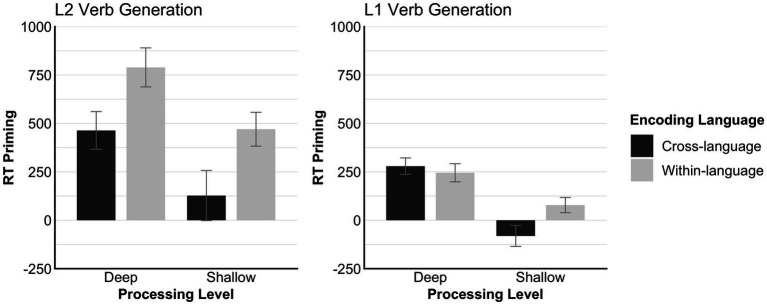
Comparison of RT priming in L2 and L1 verb generation.

**Figure 3:**
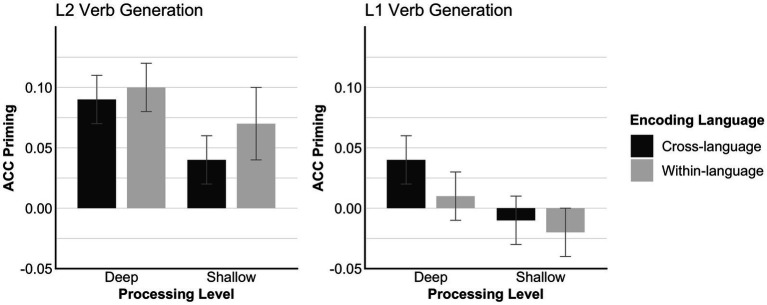
Comparison of ACC priming in L2 and L1 verb generation.

To compare the results of the two experiments, we further analyzed the self-reported data from the remaining participants. Independent-samples *t*-tests showed that there were no statistically significant differences between the two groups in terms of demographic variables, such as age, years of learning English, their listening, speaking, reading and writing in Chinese and English (*ps* > 0.05). Therefore, the participants in the two experiments were homogeneous groups, and the results of English verb generation were comparable to those of Chinese verb generation. The comparisons of RT priming and ACC priming across experiments are illustrated in Figures 2,3.

#### 3.3.1 Comparison of RT priming across experiments

A three-way repeated measures ANOVA was performed, with Task as a between-subjects factor and Encoding Language and Processing Level as within-subjects factors. The results revealed a main effect of processing level, *F* (1, 144) = 74.688, *p* < 0.001, η*_p_^2^* = 0.342, with larger priming in the deep-encoding condition (444.51 ms) than in the shallow-encoding condition (148.90 ms). The main effect of encoding language was significant, *F* (1, 144) = 18.745, *p* < 0.001, η*_p_^2^* = 0.115, with within-language priming (395.77 ms) stronger than cross-language priming (197.64 ms). The main effect of task was also significant, *F* (1, 144) = 13.637, *p* < 0.001, η*_p_^2^* = 0.087, indicating significant differences in the magnitude of repetition priming across the two tasks. The priming for L2 verb generation (462.77 ms) was substantially greater than that for L1 verb generation (130.65 ms). Finally, the effects of encoding language and task interacted, *F* (1, 144) = 8.827, *p* = 0.003, η*_p_^2^* = 0.058. Pairwise comparisons further revealed significantly greater within-language priming than cross-language priming in L2 verb generation task, uncorrected *p* < 0.001, a contrast that remained significant after Bonferroni adjustment. Nonetheless, within- and cross-language priming did not differ significantly in L1 verb generation task. Within-language priming in L2 (629.81 ms) was reliably larger than that in L1 (161.74 ms), uncorrected *p* < 0.001, and this contrast remained statistically significant after Bonferroni correction. By contrast, repetition priming in the L1-L2 condition (295.72 ms) was numerically larger than that in the L2-L1 condition (99.57 ms), uncorrected *p* = 0.070. Independent-samples *t*-tests on RT priming showed that within-language priming in L2 was considerably greater compared to that in L1 in the deep-encoding and shallow-encoding conditions, *t* (144) = 4.940, uncorrected *p* < 0.001, *t* (144) = 4.125, uncorrected *p* < 0.001. The difference between priming in L1-L2 and L2-L1 was not significant in the deep-encoding condition, *t* (144) = 1.748, uncorrected *p* = 0.083, while cross-language priming in L1-L2 was numerically greater than that in L2-L1. In the shallow-encoding condition, however, no difference was observed between priming in L1-L2 and L2-L1, *t* (144) = 1.499, uncorrected *p* = 0.136.

#### 3.3.2 Comparison of ACC priming across experiments

The analysis of ACC priming scores revealed a significant main effect of processing level, *F* (1, 144) = 16.311, *p* < 0.001, η*_p_^2^* = 0.102. Repetition priming of ACC in the deep-encoding condition was considerably larger than that in the shallow-encoding condition. The main effect of encoding language was not significant, *F* (1, 144) = 0.024, *p* = 0.878, η*_p_^2^* = 0.000. The main effect of task was significant, *F* (1, 144) = 6.851, *p* = 0.010, η*_p_^2^* = 0.045. Additionally, the effects of task and encoding language marginally interacted, *F* (1, 144) = 3.855, *p* = 0.052, η*_p_^2^* = 0.026. Pairwise comparison test further showed that priming within and across languages did not differ significantly in L2 and L1 verb generation tasks. Within-language priming in L2 was reliably greater than that in L1, uncorrected *p* = 0.002, and cross-language priming in L1-L2 condition was numerically stronger than that in L2-L1 condition, uncorrected *p* = 0.092. The results of independent-samples *t*-tests suggested that within-language priming in L2 was reliably greater compared to that in L1 in the deep-encoding condition, *t* (144) = 3.012, uncorrected *p* = 0.003, and this comparison remained statistically significant after Bonferroni correction. However, the contrast between L2 and L1 within-language priming yielded no statistically reliable effect in the shallow-encoding condition, *t* (144) = 2.500, uncorrected *p* = 0.014, because this comparison did not meet the more stringent Bonferroni-adjusted alpha criterion and was therefore non-significant after correction. The difference between priming in L1-L2 and L2-L1 was not significant in the deep-encoding condition, *t* (144) = 1.688, uncorrected *p* = 0.094, but cross-language priming in L1-L2 was numerically greater than that in L2-L1. Similarly, no statistical difference was observed between priming in L1-L2 and L2-L1 in shallow-encoding conditions, *t* (144) = 1.357, uncorrected *p* = 0.177.

## 4 General discussion

This study is the first to investigate within- and cross-language repetition priming in non-proficient Chinese-English bilinguals and to determine whether processing level modulates repetition priming, using verb generation as the production task. Significant within-language repetition priming in RTs and ACC was observed in the deep-encoding and shallow-encoding conditions, while significant cross-language repetition priming occurred only in the deep-encoding condition. The results, to a large extent, indicated the integrated conceptual representations of verbs across English and Chinese in non-proficient bilinguals. The finding aligns well with previous research on integrated conceptual repetition priming across languages for nouns (Zeelenberg and Pecher, 2003; Francis et al., 2010; Francis and Goldmann, 2011), verbs (Seger et al., 1999; de la Riva López et al., 2012) and adjectives (Taylor and Francis, 2017). It is likely that the level of overlap in conceptual representations between L1 verbs and their equivalents in the L2 differs for non-proficient bilinguals (de la Riva López et al., 2012), but this difference has not eliminated cross-language repetition priming in our bilinguals. Hence, our results provide new insights that verbs may have integrated conceptual representations across two typologically distant languages in non-proficient bilingual populations, consistent with the previous findings of integrated conceptual representations of translation-equivalent nouns in non-proficient bilinguals (Li et al., 2009).

The present study demonstrated a significant reduction of cross-language repetition priming compared to within-language repetition priming under deep encoding, indicating additional priming found in the within-language condition. This pattern of priming aligns with the predictions of the Multilink model (Dijkstra et al., 2019) and converges with prior empirical findings of conceptual repetition priming in diverse tasks in bilinguals (Hernandez and Reyes, 2002; Zhang and Wang, 2005; Francis et al., 2010; Francis and Goldmann, 2011; de la Riva López et al., 2012; Taylor and Francis, 2017; for a systematic review, see Francis, 2026). According to the triangular architecture of the Multilink model (Dijkstra et al., 2019), within-language repetition priming is probably a product of processing shared information, such as orthographic, phonetic/phonological, and semantic information (see Francis et al., 2010; Taylor and Francis, 2017; Francis, 2026). Nonetheless, cross-language priming in our study do not benefit from shared orthographic or phonological information since the translation equivalents used in the present study were not cognates. As specified in the Multilink model (Dijkstra et al., 2019), non-cognate translation equivalents exhibit minimal orthographic overlap as quantified by Levenshtein distance. Accordingly, cross-language repetitions of these verb pairs cannot receive form-based facilitation and only receive weak activation via shared semantic link, which produces attenuated cross-language repetition priming. In this study, there was significant within-language priming in RTs and ACC in the shallow-encoding condition, but no significant cross-language priming across the two experiments. Consequently, the additional priming found in the within-language condition could be ascribed to the repetition of language-specific word forms. For instance, some expected verb responses could be easily generated from the language-specific forms of English or Chinese noun stimuli (e.g., designer-design; 飞机–飞). The encoding conditions involving explicit processing of target words produced stronger priming effects in both RTs and ACC (Tsuboi et al., 2021). Moreover, the repetition of language-specific processes might also result in the larger within-language repetition priming in some cases (Francis, 2020). Within-language repetition comprises the repetition of comprehending nouns, selecting verb concepts and generating verbs, whereas cross-language repetition only involves the repetition of selecting verb concepts (de la Riva López et al., 2012), which may account for the attenuation of cross-language repetition priming. Together, the repetition of language-specific word forms or processes may account for the decrease in priming across languages relative to that within a language.

The current study also revealed that within-language repetition priming in L2 verb generation far exceeded that in L1 verb generation under deep encoding. This result supports the frequency-lag hypothesis (Gollan et al., 2011) and the predictions of stronger repetition priming in the non-dominant language for less balanced bilinguals in de la Riva López et al.’s (2012) study. Obviously, the finding that greater priming occurs in L2 than in L1 is in accord with prior research in language production (e.g., Hernandez and Reyes, 2002; Francis et al., 2003; Francis and Sáenz, 2007; Francis et al., 2008; Fan et al., 2017; Francis et al., 2022), indicating that bilinguals made slower responses and exhibited larger priming in their non-proficient language than in their proficient language. Francis et al. (2003) asserted that more difficult tasks with longer RTs and lower ACC rates benefit more from repetition due to the greater potential for improvement. Bilingual participants’ much less experience with L2 words would render these words more difficult to process, resulting in stronger benefits of repetition (Alvarez et al., 2003, p. 293). Likewise, the bilingual individuals’ more frequent use of Chinese and less exposure to English may pose greater challenges for L2 lexical processing tasks. Therefore, bilinguals would get greater benefits of repetition in the L2 verb generation task. According to the RHM (Kroll and Stewart, 1994), L2 production in non-proficient bilinguals is mediated through strong L1 lexical–conceptual links. Hence, participants’ L1 equivalents are activated as a medium when generating L2 words, allowing L2 priming to be more profitable from the overlap at the lexical and semantic levels of L1 and L2 (see, Alvarez et al., 2003). In contrast, L1 production is primarily supported by direct links between lexical and conceptual representations. Thus, the overlap of lexical priming available to L2 words is not fully reciprocated in L1 priming, leading to reduced repetition priming effects. Hernandez and Reyes (2002) also assumed that the connections between the lexical-phonological and the lemma level in the non-dominant language are much weaker than those in the dominant language. The weaker links can be enhanced by the repetition that occurs close in time (Hernandez and Reyes, 2002, p. 732). In addition, Francis (2014) attempted to explain the larger priming effect in the non-dominant language by proposing that verb generation takes longer and benefits more from repetition in the non-dominant language than in the dominant language (Francis, 2014). Notably, the stronger within-language repetition priming for L2 verbs relative to L1 verbs under deep encoding is broadly consistent with the RLA of the Multilink model (Dijkstra et al., 2019). Within this computational framework, lexical nodes of the non-dominant L2 possess markedly reduced RLA among non-proficient bilinguals. To cross the activation threshold for behavioral output, such L2 representations demand a larger magnitude of incremental activation accumulated via repeated processing, which consequently generates stronger repetition priming effects for the non-dominant language.

The present study also found asymmetric cross-language repetition priming, with priming in the L1-L2 condition being numerically larger than that in the L2-L1 condition. This result parallels previous findings of asymmetric cross-language priming observed in translation priming (e.g., Wen and van Heuven, 2017; Smith et al., 2019) and repetition priming (Zhang and Wang, 2005; Francis et al., 2014). This may reflect that the degree of conceptual-to-lemma links may substantially contribute to the asymmetric cross-language repetition priming, given that these links in L2 are much weaker than those in L1 for non-proficient bilinguals. The conceptual features cannot successfully activate the L2 lemma level, increasing the difficulty in lexical activation and word production in L2. In contrast, L1 can be much more easily activated due to the stronger links between conceptual features and L1 lemma level. Therefore, the larger repetition priming in the L1-L2 condition than that in the L2-L1 condition can be observed in the present study. Importantly, this result can also be elegantly explained by the RHM, which proposes that bilinguals initially access the L2 conceptual representation through L1 lexical representation, unless they have achieved balanced proficiency in both languages. L1 primes can efficiently activate the conceptual representations shared with L2 words, thus providing strong priming for L2 targets. Nonetheless, the concepts can only partially be activated in the L2-L1 condition. In summary, this account can be made on the claim that L1 primes can be processed more adequately than L2 primes and thus facilitate the processing of their related targets (see Smith et al., 2019, p. 158).

Finally, the results of RT priming demonstrated significant differences in repetition priming between shallow- and deep-encoding conditions. Repetition priming in the deep-encoding condition was significantly larger than that in the shallow-encoding condition. Moreover, the ACC rates in the deep-encoding condition were considerably higher compared to those in the shallow-encoding condition, further demonstrating the effects of processing level on implicit memory. The results provide compelling evidence for the view that deep processing at the encoding phase generally leads to better performance than does shallow processing (Craik and Lockhart, 1972). It can be concluded that processing level imposes significant effects on repetition priming. The results of RT priming in shallow and deep processing conditions concur with what has been reported in verb generation (Seger et al., 1999, Exp. 5), category exemplar generation (Francis et al., 2010) and antonym generation (Taylor and Francis, 2017), which support the view that deep processing leads to greater priming magnitude than shallow processing (Hamann, 1990; Mulligan et al., 1999), a pattern that holds true for languages using either alphabetic or logographic writing systems. The attenuation of repetition priming in the shallow-encoding condition relative to that in the deep-encoding condition somehow provides a better understanding of the transfer-appropriate processing account, that is, the verb generation task in the present study was conceptual in essence, leading to a reduction of priming magnitude in the shallow processing condition (see Taylor and Francis, 2017). This observation is in line with prior evidence suggesting that repetition priming exerts a robust facilitative effect on conceptual processing (Decuyper et al., 2026). This study revealed significant cross-language priming only in the deep-encoding condition rather than in the shallow-encoding condition, indicating the importance of conceptual processing in cross-language repetition priming. This finding is consistent with the results of prior studies on concrete nouns (Francis et al., 2010) and adjectives (Taylor and Francis, 2017). In addition, significant within-language repetition priming occurred in the shallow-encoding condition. This result is congruent with previous single-language research (e.g., Hamann, 1990; Mulligan et al., 1999; Seger et al., 1999), but contrary to the finding reported in a bilingual study (Taylor and Francis, 2017). An alternative interpretation for the difference might lie in the shallow processing tasks performed (vowel-counting task vs. orthographic judgment task). Sufficient lexical processing may not be guaranteed in a vowel-counting task (Newell and Andrews, 2004) to detect a priming effect, while the latter may render lexical processing more effective. Based on the “levels of processing” framework, deep processing typically demands greater cognitive effort than shallow processing (Craik and Lockhart, 1972). Consequently, the shared component processes involved in deep processing are more cognitively demanding and difficult than those in shallow processing, which in turn gives rise to a larger magnitude of repetition priming under the deep processing condition (see Francis et al., 2025).

## 5 Conclusion

The present study investigated within- and cross-language repetition priming among non-proficient Chinese-English bilinguals in the context of verb generation. The results can be summarized as follows. Firstly, non-proficient bilinguals showed substantial within-language priming in the deep-encoding and shallow-encoding conditions, while significant cross-language priming occurred only in the deep-encoding condition, predominantly revealing the integrated conceptual representations of verb translation equivalents in English and Chinese. Secondly, within-language priming was significantly larger than cross-language priming in the deep-encoding condition. Repetition priming in L2 was reliably greater than that in L1 in the deep-encoding condition, and cross-language priming in the L1-L2 condition was stronger than that in the reverse direction. Thirdly, priming in the deep-encoding condition was substantially larger than that in the shallow-encoding condition, exhibiting a levels-of-processing effect. Overall, the study broadly indicated that conceptual representations of verbs are integrated across two distant languages for non-proficient bilinguals.

In conclusion, the current study addressed some fundamental issues in implicit memory for verbs and added to the literature on conceptual representations of bilingual memory in non-proficient bilinguals. Yet, the study only used behavioral methods and isolated words. Future studies could employ cognitive neuroscience techniques, such as ERP and fMRI, to explore the neural correlates and brain activity in word production with context using a repetition priming paradigm, providing a more comprehensive profile of the nature of bilingual lexical and conceptual representations. Moreover, nouns can elicit their semantically associated verbs, whereas the positional probability of such verbs was inconsistent across experimental stimuli in this study. It remains to be further investigated whether word order preference (i.e., the probability of verbs occurring before or after nouns) significantly affects experimental results. Another limitation is that participants were classified as non-proficient bilinguals solely by virtue of self-reported language proficiency and CET-4 performance, with no objective assessments of vocabulary size, lexical access, reading and speaking proficiency. Future research should adopt more rigorous and objective measures to differentiate between proficient and non-proficient bilinguals.

## Data Availability

The datasets presented in this study can be found in online repositories. The names of the repository/repositories and accession number(s) can be found in the article/Supplementary material.
