# Supplementary material for: Within- and cross-language repetition priming in verb generation: evidence from non-proficient Chinese-English bilinguals
Source: Front Psychol. 2026 Jul 29;17:1857807. doi: 10.3389/fpsyg.2026.1857807 (PMC13461605; doi:10.3389/fpsyg.2026.1857807)
Supplement: Supplementary file 1 [file Table_1.DOCX]

Stimuli Used in the Experiment

| **English Nouns** | **Expected Verbs** | **Chinese Nouns** | **Expected Verbs** |
| --- | --- | --- | --- |
| food | eat | 食物 | 吃 |
| guitar | play | 吉他 | 弹 |
| bike | ride | 自行车 | 骑 |
| wine | drink | 酒 | 喝 |
| music | listen | 音乐 | 听 |
| movie | watch | 电影 | 看 |
| oxygen | breathe | 氧气 | 呼吸 |
| chair | sit | 椅子 | 坐 |
| bed | sleep | 床 | 睡 |
| experiment | do | 实验 | 做 |
| ticket | buy | 车票 | 买 |
| mountain | climb | 山 | 爬 |
| story | tell | 故事 | 讲 |
| mirror | look | 镜子 | 照 |
| heart | beat | 心脏 | 跳 |
| hair | cut | 头发 | 剪 |
| pencil | write | 铅笔 | 写 |
| telephone | call | 电话 | 打 |
| foot | walk | 脚 | 走 |
| cleaner | clean | 清洁工 | 扫 |
| bathroom | wash | 浴室 | 洗 |
| tool | use | 工具 | 用 |
| spokesman | speak | 发言人 | 发言 |
| actor | act | 演员 | 演 |
| page | turn | 页 | 翻 |
| student | study | 学生 | 学习 |
| designer | design | 设计师 | 设计 |
| bell | ring | 铃 | 响 |
| car | drive | 车 | 开 |
| attention | pay | 注意力 | 集中 |
| pool | swim | 水池 | 游 |
| waiter | serve | 服务员 | 服务 |
| athlete | run | 运动员 | 跑 |
| rubbish | throw | 垃圾 | 扔 |
| airplane | fly | 飞机 | 飞 |
| accident | happen | 事故 | 发生 |
| clothing | wear | 衣服 | 穿 |
| list | make | 清单 | 列 |
| baby | cry | 婴儿 | 哭 |
| picture | draw | 图片 | 画 |
| thief | steal | 小偷 | 偷 |
| mother | love | 母亲 | 爱 |
| mixture | mix | 混合物 | 混合 |
| professor | teach | 教师 | 教 |
| environment | protect | 环境 | 保护 |
| wind | blow | 风 | 吹 |
| length | measure | 长度 | 量 |
| hen | lay | 母鸡 | 下蛋 |
| task | finish | 任务 | 完成 |
| rule | obey | 规则 | 遵守 |
